# Hypoxia-Induced Endothelial Damage and Microthrombosis in Myocardial Vessels of Newborn Landrace/Large White Piglets

**DOI:** 10.1155/2014/619284

**Published:** 2014-03-04

**Authors:** Armando Faa, Theodoros Xanthos, Vassilios Fanos, Daniela Fanni, Clara Gerosa, Pietro Pampaloni, Maria Elena Pais, Gavino Faa, Nicoletta Iacovidou

**Affiliations:** ^1^Department of Pathology, University of Cagliari, Via Ospedale 46, 09100 Cagliari, Italy; ^2^Medical School, National and Kapodistrian University of Athens, 100 Athens, Greece; ^3^Department of Paediatrics, Neonatal Intensive Care Unit, Puericulture Institute and Neonatal Section, University of Cagliari, 09100 Cagliari, Italy; ^4^2nd Department of Obstetrics and Gynecology, Neonatal Division, Medical School, National and Kapodistrian University of Athens, 100 Athens, Greece

## Abstract

*Objective.* Evaluating the presence of endothelial changes in myocardial vessels in an experimental model of hypoxia and resuscitation in newborn piglets. *Methods.* Fifty male Landrace/Large White neonatal piglets were studied: ten of them were allocated in group A (control group, SHAM-operated). In group B (forty animals, experimental group) normocapnic hypoxia was induced by decreasing inspired concentration of O_2_ to 6%–8%. When the animals developed bradycardia or severe hypotension, reoxygenation was initiated. The animals of group B were allocated in 4 subgroups of 10, according to the concentration of O_2_ they were resuscitated with (groups 1, 2, 3, and 4 received 18%, 21%, 40%, and 100% O_2_, resp.). *Results.* Control group animals did not show any significant endothelial lesions. Contrarily, endothelial lesions were detected in all experimental group cases. When these lesions were analyzed in the different heart zones, no significant difference in their incidence was observed; analyzing the frequency in the animals of the 4 subgroups, only microthrombosis showed a higher frequency in animals in groups 4 and 3. *Conclusions.* Endothelial damage represents a diffuse pathological feature in the myocardial vessels of piglets subjected to normocapnic hypoxia and resuscitation suggesting a possible role of hyperoxygenation in aggravating endothelial damage.

## 1. Introduction

Birth asphyxia is a major issue for health care systems, affecting around 2–4 per 1,000 full-term newborns in developed countries [1]. Perinatal asphyxia causes injury to the central nervous, the cardiovascular, the renal, and the respiratory system and to the hematologic organs, the adrenals, and the liver [2, 3]. Multiple organ dysfunction syndrome (MODS) represents the most severe complication in neonates with perinatal asphyxia admitted to neonatal intensive care units (NICUs) and a major cause of short- and long-term morbidity [4–6]. The mechanism of cellular injury in hypoxic/ischemic MODS and that of acute heart injury have not been completely clarified yet. Recent studies suggested that vascular changes might play a relevant role in acute kidney injury [7, 8] and in acute lung injury [9]. The following sequence of events leading to hypoxia-/asphyxia-induced MODS has been hypothesized: first failure of the gut barrier, endotoxemia, and release of proinflammatory cytokines, including IL-1b, TNF alpha, IL-6, and ETX, lead to upregulation of adhesion molecules in the vascular bed of multiple organs. This universal endothelial injury, characterized by endothelial swelling and apoptosis, results in generalized capillary leak. This creates generalized edema and activation of intrinsic inflammatory cells in different organs, producing in this way multiple organ failure [6, 10, 11].

As previously reported by our group the histological study of the myocardial cells in this experimental piglet model of normocapnic hypoxia/reoxygenation showed significant cardiac changes after hypoxia and reperfusion [12, 13]. This study aimed at identifying endothelial changes in the myocardium of newborn piglets following hypoxia, in order to examine if the endothelial cells represent a target of hypoxic injury in the cardiac vascular structures.

## 2. Materials and Methods

Fifty male Landrace/Large White piglets aged 1–4 days and weighing 2.3–3.8 kg were the study subjects; 10 of them served as controls (group A) and 40 of them were included in the experimental group (group B). The animals were obtained from the same breeding unit (N. Validakis, Koropi, Greece) and were transported individually to the laboratory (Experimental-Research Center ELPEN) on the day of experimentation; feeding was not required at the research facility.

The experimental protocol was approved by the General Directorate of Veterinary Services (Permit number 404/21-04-09) according to Greek Legislation on scientific and experimental procedures (Presidential Decree 160/1991, in compliance with the Directive 86/609/EEC).

Animals were initially sedated with a single intramuscular injection of ketamine 10 mg/kg (Narketan, Vétoquinol UK Ltd.) and midazolam 0.5 mg/kg (Dormicum, Hoffmann-La Roche, Germany) as previously described [13]. The marginal auricular vein was catheterized with a 24G catheter (Jelco R., Smiths Medical, N. Papapostolou SA, Athens, Greece) and anesthesia was induced with propofol 1 mg/kg (Diprivan, AstraZeneca) and fentanyl 10 *μ*g/kg (Fentanyl, Janssen-Cilag). The piglets were intubated with a 3.0 or 3.5 mm endotracheal tube (Portex, Smiths Medical, UK) as appropriate. Correct placement of the endotracheal tube was confirmed by auscultation and capnography (Datex Engstrom, Type TC 200-22-01 Instrumentarium Corp., Helsinki, Finland). Infusion of 10 mL/kg/h NaCl 0.9% and 5 mL/kg/h dextrose in water 5% was initiated to prevent dehydration and hypoglycemia. Noninvasive continuous monitoring included recording of heart rate (HR), electrocardiogram (ECG), saturation of oxygen by pulse oximeter (SpO_2_), and rectal temperature (Matron, BPM 1000, VET, ET Medical Devices Spa). Body temperature was maintained at 38 ± 1°C with the aid of a table heating pad and an overhead heating lamp. Intravenous (iv) boluses of fentanyl 20 *μ*g/kg and cisatracurium 0.2 mg/kg (Nimbex, Abbott), as well as prophylactic antibiotic cefuroxime 25 mg/kg (Zinacef, GlaxoSmithKline), were administered after which the animals were mechanically ventilated (Soxil, Soxitronic, Felino, Italy) with a tidal volume (*V*_*T*_) of 10–15 mL/kg, pressure of 19 cm H_2_O, and respiratory rate of 30–40 breaths per minute (aiming at maintaining end-tidal CO_2_ (ETCO_2_) of 35–45 mm Hg). The fraction of inspired oxygen (FiO_2_) was adjusted between 0.21 and 0.25 in order to maintain SpO_2_ of 90–95%. Anesthesia was maintained with the infusion of 8–10 mg/kg/h propofol and boluses of 10 *μ*g/kg fentanyl and 0.15 mg/kg cisatracurium.

Subsequently, the right internal jugular vein and carotid artery were catheterized, with single-lumen fluid-filled silicone catheters (S1UVC5.0, NeoCare; Klein-Baker Medical Co., San Antonio, TX, USA). The catheters were secured and connected to external transducers (Transpac, Abbott Critical Care Systems, USA), for continuous monitoring of central venous pressure (CVP), systolic (SP), mean (MP), and diastolic pressure (DP) of the carotid artery. Following catheterization, the incision was sutured and covered with sterile warm gauzes to prevent heat loss. The animals were stabilized for 30 minutes and baseline arterial blood samples were obtained.

Animals in group A were SHAM-operated and were handled according to the protocol. They did not undergo hypoxia, nor did they receive resuscitation.

In animals of group B, hypoxia was induced by decreasing the inspired FiO_2_ to 0.06–0.08, while the other settings of the ventilator remained unchanged. Close monitoring was carried out to detect signs of hemodynamic instability, defined as either bradycardia (HR < 60 beats per minute) or severe hypotension (MP < 15 mm Hg). The time required for these conditions to occur was recorded using a digital stopwatch. As soon as hemodynamic compromise occurred, arterial blood samples were obtained, to confirm hypoxemia (pO_2_ 30–50 mm Hg), and resuscitation efforts were initiated.

Animals were resuscitated with different oxygen concentrations according to allocation and subdivided into four groups: groups 1, 2, 3, and 4 received 18%, 21%, 40%, and 100% O_2_, respectively, and at the same concentration, until HR and MAP returned to 90% of their baseline levels. The effectiveness of resuscitation efforts was assessed every 30 seconds. If despite oxygen administration for 30 seconds the HR did not increase, oxygenation was administered for 30 more seconds. If HR did not increase after two cycles (30 seconds/cycle) of oxygenation, chest compressions were applied at a rate of 3 compressions: 1 ventilation for 30 seconds. Adrenaline (1 : 10,000 dilution), at a dose of 10 mcg/kg *via* the auricular venous catheter, was administered if one cycle of chest compressions failed to increase the HR. As soon as these hemodynamic parameters returned to baseline values, the piglets were ventilated under anesthesia for 30 more minutes prior to collecting arterial blood for blood gas analysis. Endpoints of the experiment were either persisting asystole despite 10 minutes of cardiopulmonary resuscitation, or return of the hemodynamic parameters to baseline values. The animals were then humanely subjected to euthanasia by slow intravenous infusion of sodium thiopental (Pentothal, Hospira Enterprises BV, The Netherlands) at a dose of 30 mg/kg. Necropsy followed examination of possible injury or abnormality.

Multiple cardiac tissue samples were collected *in toto*, reduced, fixed in 10% formalin, routinely processed, and paraffin-embedded; the initial block was cut into 6-7 blocks about 2-3 mm wide that were previously deparaffinized and hydrated to water. They were then colored with hematoxylin for 15 minutes, washed in running tap water for 20 minutes, and counterstained with eosin for 15 seconds to 2 minutes. Finally slides were dehydrated in 95% absolute alcohol and cleared in xylene. A pathologist blinded to the animal outcome assessed all hematoxylin-eosin (H&E) slices. The variables are expressed as ratio between the number of cases with the specific change and the total number in each group. Repeated Student's *t*-test was used to evaluate any possible statistically significant differences for each elementary lesion found at pathology between the control group and the experimental subgroups.

## 3. Results

No histological changes were detected in the endothelium of arteries or veins in the myocardium samples of the control animals.

**Figure 1:**
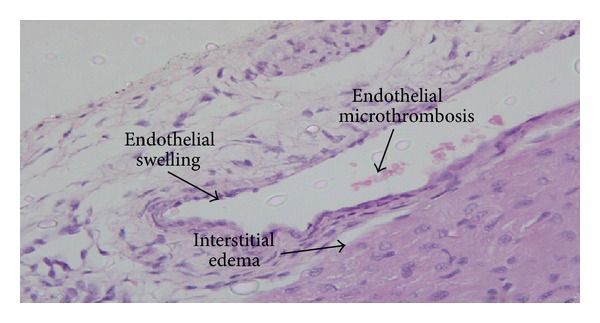
Endothelial damage, edema, and hypoperfusion in heart vessels following asphyxia.

**Figure 2:**
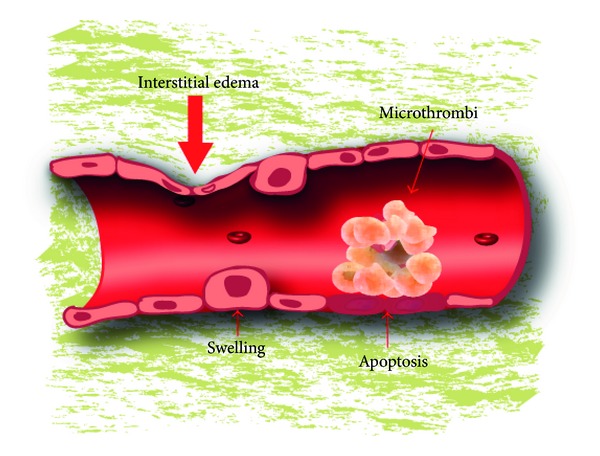
Model of principal endothelial lesions observed.

Contrarily, the histological examination of H&E-stained sections in the experimental groups revealed pathological changes in the endothelium of cardiac veins and arteries in all the heart samples studied (Figures 1 and 2). Endothelial swelling, endothelial detachment, endothelial loss, edema, endothelial apoptosis, and thrombosis were lesions identified in the intracardiac arteries and veins of multiple heart regions (subepicardial area, intracardiac region, and the subendocardial zone) (Table 1). When endothelial lesions were compared among animals in subgroups 1–4, no significant differences were observed among groups, regarding the frequency and the intensity for the majority of vascular endothelial lesions studies. Only microthrombosis showed marked differences among the groups (Table 2) being significantly more frequent, especially in the intracardiac region, in groups 3 and 4 compared to groups 1 and 2 (*P* < 0.001). On the contrary, marked differences in the degree of vascular lesions were observed among animals of the same group.

## 4. Discussion

Several experimental studies have recently provided new insights into cellular and molecular events occurring in tissues following asphyxia. Microvascular dysfunction and endothelial cell damage have been identified as factors playing a key role not only in single organ dysfunction after asphyxia but also in the development of multiple organ failure in newborns affected by hypoxia [12]. In an *in vitro* system of coronary endothelial cells, energy depletion was shown to be responsible for endothelial hyperpermeability [14]. The increase in endothelial permeability, responsible for interstitial edema, was subsequently identified in structural modifications in cell-to-cell adhesion and in the tight junctions of the endothelium [15] up to their complete loss [16]. The disruption of adherence junctions was shown to be responsible for the increased permeability of vascular endothelium, followed by leakage from the vascular bed to the surrounding tissues resulting in interstitial edema [17]. Another factor contributing to perfusion impairment of multiple organs after asphyxia is endothelial cell swelling [18] which may cause shrinking of the vascular lumen by external pressure due to the interstitial edema [19]. In addition, endothelial cell injury might induce a procoagulative response, leading to microthrombosis and contributing to vascular obstruction and hypoperfusion of multiple tissues [20].

In the present study, we have shown that the endothelial damage may represent an important component of the response to hypoxia in the vessel of the myocardium of newborn piglets undergoing asphyxia. We have recently reported that interstitial edema represented the most frequent pathological change in cardiac cells of asphyxiated piglets, being reported in 37 out of 40 heart samples analyzed [11]. With the present data we suggest that the cause of interstitial edema may be identified in endothelial damage. Endothelial changes were found both in arteries and in veins in the subepicardial, in the intracardiac, and in the subendocardial zones. The most frequent endothelial lesion observed was endothelial swelling, detachment, loss, and edema (100%), followed by apoptosis (73.3%) of endothelial cells and thrombosis (57.6%). Taken together, all these relevant endothelial changes may be considered responsible for the hyperpermeability of heart vessels, leading to interstitial edema, and for the hypoperfusion of cardiomyocytes, mainly related to microthrombosis. An interindividual variability among experimental animals was also found, regarding the prevalence of the vascular damage, evidenced by marked differences in the degree of vascular lesions observed among animals of the same group, receiving the same experimental treatment.

In subgroups 1–4, based on the different oxygen concentrations used for resuscitation, no significant differences were observed among groups regarding the frequency and the intensity of the majority of vascular endothelial lesions studies. Only microthrombosis showed marked differences among the groups, appearing significantly more frequent in groups 3 and 4.

Interestingly, the highest degree of endothelial damage was observed in the animals of subgroup 4. This finding may suggest a role for oxygen treatment in worsening endothelial damage, with all the negative consequences in heart cell function.

## 5. Conclusions

Our study shows that endothelial damage represents a relatively diffuse pathological feature in the heart of newborn piglets submitted to normocapnic hypoxia and resuscitation. Endothelial cell swelling, apoptosis of endothelial cells, and microthrombosis are the most frequent lesions observed in all the 40 newborn piglets examined in the present study. The finding of the vascular lesions in all the heart zones underlines the clinical significance of the cardiac endothelial damage, which appears as the main factor responsible for interstitial edema and for hypoperfusion of cardiomyocytes under hypoxic/ischemic conditions. Further studies are needed focusing on the cellular mechanism which may be responsible for hypoxia-induced endothelial injury, in order to allow neonatologists to promptly intervene towards prevention of cardiac damage in asphyxiated neonates.

## Figures and Tables

**Table 1:** Occurrence of endothelial lesions in animals of subgroups 1–4.

	Subepicardic						Intracardiac						Endocardic					
	SW	DTC	Loss	AP	TR	ED	SW	DTC	Loss	AP	TR	ED	SW	DTC	Loss	AP	TR	ED
1	10/10	10/10	10/10	7/10	4/10	10/10	10/10	10/10	10/10	9/10	2/10	10/10	10/10	10/10	10/10	2/10	5/10	10/10
2	10/10	10/10	10/10	5/10	3/10	10/10	10/10	10/10	10/10	5/10	4/10	10/10	10/10	10/10	10/10	7/10	5/10	10/10
3	10/10	10/10	10/10	10/10	7/10	10/10	10/10	10/10	10/10	10/10	6/10	10/10	10/10	10/10	10/10	8/10	7/10	10/10
4	10/10	10/10	10/10	10/10	6/10	10/10	10/10	10/10	10/10	8/10	9/10	10/10	10/10	10/10	10/10	7/10	9/10	10/10

SW: swelling; DTC: detachment; Loss: endothelial loss; AP: apoptosis; TR: microthrombosis; ED: edema.

**Table 2:** Occurrence of microthrombosis in animals of subgroups 1–4.

Group	Subepicardial	Intracardiac	Subendocardial	Total score
1	4/10	2/10	4/10	10/40
2	3/10	4/10	5/10	12/40
3	7/10	5/10	7/10	19/40
4	6/10	9/10	5/10	20/40
